# Association of Vaginal Progesterone Treatment With Prevention of Recurrent Preterm Birth

**DOI:** 10.1001/jamanetworkopen.2022.37600

**Published:** 2022-10-31

**Authors:** David B. Nelson, Ashlyn Lafferty, Chinmayee Venkatraman, Jeffrey G. McDonald, Kaitlyn M. Eckert, Donald D. McIntire, Catherine Y. Spong

**Affiliations:** 1 Department of Obstetrics and Gynecology, University of Texas Southwestern Medical Center at Dallas, Dallas; 2 Department of Obstetrics, Parkland Health, Dallas, Texas; 3 Center for Human Nutrition, University of Texas Southwestern Medical Center at Dallas, Dallas; 4 Department of Molecular Genetics, University of Texas Southwestern Medical Center at Dallas, Dallas

## Abstract

**Importance:**

Preterm birth (PTB) is the leading cause of infant morbidity and mortality worldwide. It has been suggested that vaginal progesterone (VP) treatment may reduce the recurrence of PTB.

**Objective:**

To evaluate the association of VP treatment with prevention of recurrent PTB among patients with a singleton pregnancy.

**Design, Setting, and Participants:**

This prospective, observational cohort study, set in a public health care system for inner-city pregnant patients, enrolled patients with prior spontaneous PTB (gestational age, ≤35 weeks) receiving VP from May 15, 2017, to May 7, 2019. Patients who delivered between 1998 and 2011 served as a referent cohort matched 3:1 for obesity, race and ethnicity, and individual specific preterm birth history. Statistical analysis was performed from August 19, 2021, to September 2, 2022.

**Exposure:**

Patients received 90 mg of vaginal progesterone, 8%, nightly, initiated between 16 weeks and 0 days and 20 weeks and 6 days of pregnancy until 36 weeks and 6 days of pregnancy or delivery.

**Main Outcomes and Measures:**

The primary outcome was overall rate of recurrent PTB at 35 weeks or less of patients given VP compared with the 3:1 matched untreated historical controls. Secondary outcomes included assessment of PTB according to adherence (≥80% completing scheduled doses), duration of pregnancy relative to index gestational age, progesterone blood levels, and outcomes for those who declined VP.

**Results:**

A total of 417 patients (mean [SD] age, 30.4 [5.9] years; 64 Black patients [15.3%]; 272 [65.2%] with a body mass index of ≥30) received VP and were matched with 1251 controls (mean [SD] age, 28.8 [5.7] years; 192 Black patients [15.3%]; 816 [65.2%] with a body mass index of ≥30). The overall rate of recurrent PTB was 24.0% (100 of 417; 95% CI, 20.0%-28.4%) for the VP cohort compared with 16.8% (1394 of 8278) expected in the matched historical controls. Adherence was not associated with lower rates of recurrent PTB compared with nonadherence (odds ratio, 0.87 [95% CI, 0.51-1.41]). The mean difference between historical matched controls and those using VP was 0.2 weeks (95% CI, −1.4 to 1.0 weeks) without improvement in the interval of recurrent PTB after the implementation of VP (*P* = .73). Progesterone blood levels for patients who were adherent compared with those who were nonadherent were not significantly different at either 24 or 32 weeks (24 weeks: 99 ng/mL [95% CI, 85-121 ng/mL] vs 104 ng/mL [95% CI, 89-125 ng/mL]; *P* = .16; 32 weeks: 200 ng/mL [95% CI, 171-242 ng/mL] vs 196 ng/mL [95% CI, 155-271 ng/mL]; *P* = .69).

**Conclusions and Relevance:**

This cohort study of patients with a current singleton pregnancy suggests that VP was not associated with a reduction in recurrent PTB.

## Key Points

**Question:**

Is vaginal progesterone treatment associated with reduced risk of recurrent spontaneous preterm birth (gestational age, ≤35 weeks) among patients with singleton pregnancies?

**Findings:**

In this cohort study of 417 pregnant patients with previous preterm births, use of vaginal progesterone was not associated with a reduction in preterm birth when accounting for both frequency and severity of prior preterm births.

**Meaning:**

This study suggests that, for patients with prior preterm birth, vaginal progesterone treatment was not associated with a reduction in recurrence of preterm birth.

## Teaser

This cohort study evaluates the association of vaginal progesterone treatment with prevention of recurrent preterm birth among patients with a singleton pregnancy.

## Introduction

Preterm birth (PTB) is a public health concern with well-documented consequences for the infant, family, and society.^1^ Identified as the leading cause of death among children younger than 5 years of age, PTB affects nearly 15 million births worldwide and remains the foremost issue in obstetrics.^1,2^ Although the burden of PTB is clear, identifying strategies to reduce PTB has been challenging. Spontaneous PTB represents a syndrome with multiple causes; however, one of the strongest risk factors for recurrence is a history of PTB.^3,4,5,6^

It is currently proposed that parturition follows functional progesterone withdrawal; thus, progestogens—either 17-alpha hydroxyprogesterone caproate (17OHP-C) or vaginal progesterone (VP)—have been at the forefront of efforts to prevent PTB. Currently, both the American College of Obstetricians and Gynecologists and the Society for Maternal-Fetal Medicine support using progestogen therapy for the prevention of recurrent PTB for select patients with singleton pregnancies.^7,8^ In a 2017 prospective study of 17OHP-C for 430 patients with a prior delivery at 35 weeks or less gestational age, treatment did not result in a reduction of recurrent PTB compared with a historical cohort of untreated patients with similar PTB profiles.^6^ Thereafter, the postmarketing follow-up randomized double-blind, placebo-controlled clinical trial (PROLONG [17-OHPC to Prevent Recurrent Preterm Birth in Singleton Gestations]) also did not demonstrate efficacy of 17-OHPC among patients with a singleton pregnancy and prior spontaneous PTB.^9^

The purpose of this study is to describe the introduction of VP treatment into our practice for the prevention of recurrent PTB among patients in an urban, inner-city hospital. The primary aim was to evaluate the association of VP with the prevention of birth at 35 weeks or less using each patient—and their specific history of PTB—as the benchmark to measure response. We hypothesized that VP treatment would reduce recurrent PTB among patients compared with matched historical controls with similar number and frequency of prior PTBs. We also examined therapy and clinical outcomes associated with medication adherence, severity of recurrent PTB, and progesterone blood levels.

## Methods

This study was approved by the institutional review board of the University of Texas Southwestern Medical Center. Because VP was deployed as the standard of care during the study period, verbal informed consent to participate was obtained with the option to opt out. Written informed consent was obtained for study participants who had blood samples obtained. This report followed the Strengthening the Reporting of Observational Studies in Epidemiology (STROBE) reporting guideline for cohort studies.

Parkland Health serves underresourced patients of Dallas County and has developed a neighborhood-based, administratively and medically integrated public health care system for inner-city pregnant patients.^10,11^ All patients are assigned to a neighborhood clinic for antenatal care. On enrollment into prenatal care, patients with a history of PTB are referred to a dedicated clinic centrally located at Parkland Hospital. This high-risk prenatal clinic is staffed by maternal-fetal medicine faculty and fellows from the University of Texas Southwestern Medical Center. Criteria for referral to this clinic include singleton pregnancy and prior spontaneous PTB or rupture of membranes between 20 weeks and 0 days and 35 weeks and 0 days gestational age.

This was an inception, cohort study design wherein VP was offered to all patients in this clinic beginning May 15, 2017. The outcome of interest was recurrent PTB among patients treated with VP compared with a historical cohort before any progestogen was offered. Every patient underwent a detailed obstetric history that included the number of confirmed previous births, gestational age at PTB, reason(s) for PTB, birth weight, and perinatal outcome.^6^ Patients with a medically indicated PTB—such as pregnancy-related hypertension—were excluded. For those with more than 1 pregnancy during the study period, the first pregnancy outcome was used for analysis. Because PTB risk differs by race and ethnicity, this information was obtained by research staff using patient self-report. Gestational age was based on the date of the last menstrual period with sonographic verification. Data on prior obstetric history was obtained from an obstetric database with maternal and infant outcomes for all deliveries.^12^ Patients meeting inclusion criteria with prior spontaneous PTB at 35 weeks or less owing to either spontaneous labor or rupture of membranes during any prior pregnancy were offered VP. Therapy was initiated between 16 weeks and 0 days and 20 weeks and 6 days gestational age using 90 mg of VP, 8%, nightly (provided by the hospital pharmacy) until 36 weeks and 6 days or delivery. Information sheets for medication use were provided to the patients in the clinic.

### Assessment of Clinical Effectiveness: Primary and Secondary Outcomes

The primary outcome was the overall rate of recurrent PTB at 35 weeks or less for patients given VP compared with the 3:1 matched untreated historical cohort. This analysis was conducted using an intention-to-treat principle. This gestational age was chosen because it has been recognized by the US Food and Drug Administration as a clinically relevant outcome of interest and is identical to a prior report as well as the PROLONG trial.^6,9,13^ Cervical length was available for a limited number of patients; however, too few cases were available to draw meaningful conclusions.

The analysis also included examination of VP use for patients with differing PTB and term birth histories. Because an individual patient’s risk for recurrent PTB is associated with the past number and sequence of PTB(s), we analyzed the rate of recurrence according to the number of prior PTBs, as well as the sequence of both preterm and term infants.^4,6^ For example, a risk of recurrent PTB for a patient with 3 pregnancies and 2 live births with a prior PTB followed by a term birth differs from the risk for a patient with a prior term birth followed by PTB.

Patients who delivered at Parkland Hospital between January 1, 1998, and December 31, 2011, before implementation of either VP or 17OHP-C were used as a referent cohort. Patients were matched 3:1 with untreated historical controls compared with VP for obesity (body mass index [BMI], ≥30 [calculated as weight in kilograms divided by height in meters squared]), race and ethnicity, and individual specific PTB history—using both the number and the sequence of previous PTBs to minimize asymmetry of recurrent PTB risk.^6,12^ The match was an exact greedy match for obesity with weight at delivery, non-Hispanic Black race, and prior PTB pattern as preterm delivery of the 1 prior delivery for 1 live birth, all 3 possible patterns for 2 live births (both births preterm, last birth preterm, and first birth preterm), and for 2 patterns for 3 live births (last birth preterm and last birth not preterm). These factors were chosen because of the known strength of association with recurrent PTB.^6^

There were 5 secondary outcomes. First, we assessed whether the rate of PTB recurrence was associated with adherence with VP treatment. Adherence was assessed by trained research nurses recording individual patient doses at 2-week intervals using a process similar to the Morisky Medication Adherence Scale assessment.^14^ Individual patient doses were calculated according to anticipated doses up to 36 weeks and 6 days gestational age or delivery. Patient report was verified with pharmacy dispensed medication. Adherence was defined as 80% or more of completed doses.^15^

Second, we sought to compare a specific patient’s gestational age at the time of a prior PTB with the gestational age at delivery when treated with VP.^5,6^ Put another way, we sought to evaluate the association of VP treatment with the length of pregnancy.^16^ To do this, we compared the change in duration of pregnancy measured in weeks of gestation for patients with recurrent PTB after VP treatment with untreated patients who had previously delivered at our hospital.^6^

Third, we sought to ascertain whether PTB was associated with progesterone levels among patients treated with VP. Plasma progesterone levels were measured at 24 and 32 weeks, coinciding with routine blood samples obtained for prenatal care. Progesterone was measured based on methods described by Honda et al.^17^ The association of progesterone blood levels with PTB was also compared with the rate of adherence. Quantitative measurement was performed via high-performance liquid chromatography–mass spectrometry with atmospheric pressure chemical ionization. All analyses were conducted by a coinvestigator (J.G.M.).

Fourth, we compared pregnancy outcomes for patients who declined any use of progestogens during the study with those treated with VP to address selection bias of the study cohort. Pregnancy outcomes for patients who declined progestogen use during the study period were analyzed for both PTB history and gestational age at delivery. This analysis included comparison of specific PTB profiles according to patient history. Fifth, we included a comparison of pregnancy outcomes during the period of 17OHP-C use (2012-2017) with the contemporary study period with VP use.

### Statistical Analysis

Statistical analysis was performed from August 19, 2021, to September 2, 2022. The rate of recurrent PTB at 35 weeks or less in the historical obstetric population was 16.8% (1394 of 8278) when progestogen was not in use.^5^ This rate was used to calculate the sample size. A sample size of 413 patients was estimated for 80% power to detect a one-third reduction in recurrent PTB (from 16.8% to 11.2%) using a 2-sided, 1-sample binomial test of size .05 (α = .05). Recurrence rates according to the prior number and sequence of specific histories of PTB and term births were also based on the Parkland obstetric population prior to progestogen use. For 2-group comparisons, the Pearson χ^2^ test was used for categorical outcomes and the *t* test for continuous outcomes. When categories included strata—as with the PTB profile—the Cochran-Mantel-Haenszel χ^2^ test was used to evaluate the association adjusted for strata. Odds ratios (ORs) and 95% CIs are presented for effect size with this adjustment. Simple linear regression was used to evaluate the association between 2 continuous measures, such as gestational age at delivery and adherence. For the primary outcome, the Cochran-Mantel-Haenszel test was adjusted for the case-control structure. Logistic regression was used to estimate the association between a categorical outcome (ie, recurrent PTB) and continuous independent risk, such as percentage adherence. Statistical analysis was performed using SAS, version 9.4 (SAS Institute Inc).

## Results

Between May 15, 2017, and May 7, 2019, 417 study patients (mean [SD] age, 30.4 [5.9] years; 64 Black patients [15.3%]; 332 Hispanic patients [79.6%]; 272 [65.2%] with a BMI of ≥30) were included and were matched with 1251 controls (mean [SD] age, 28.8 [5.7] years; 192 Black patient [15.3%]; 816 [65.2%] with a BMI of ≥30) (Table 1). A total of 2883 patients were screened, and 649 were eligible for VP treatment. Those with entry to care at beyond 21 weeks (n = 968), cerclage (n = 53), multiple gestation (n = 21), incarceration (n = 56), and with pregnancy losses before 20 weeks (n = 65) were excluded. Of the 649 patients eligible, 452 agreed to receive VP, and the other 197 declined. Of 452 patients, 10 (2.2%) were lost to follow-up, 7 had stillbirths, and 18 had more than 1 pregnancy within the study period wherein the first of the 2 pregnancies was used for analysis. Thus, 417 eligible patients with prior PTB at 35 weeks or less were treated with VP and delivered with complete data for the study cohort.

**Table 1.** Selected Demographic and Clinical Characteristics of Study Cohort With 3:1 Matched Historical Controls

Characteristic	No. (%)		*P* value
	Cases (n = 417)	Controls (n = 1251)	
Age, mean (SD), y	30.4 (5.9)	28.8 (5.7)	<.001
Race and ethnicity			
African American or Black^a^	64 (15.3)	192 (15.3)	.09
Hispanic or Latinx	332 (79.6)	990 (79.1)	
White	12 (2.9)	58 (4.6)	
Other^b^	9 (2.2)	1 (0.08)	
Obesity^a^^,^^c^	272 (65.2)	816 (65.2)	>.99
Preterm profile^b^			
1 Live birth	122 (29.2)	366 (29.3)	>.99
2 Live births			
Most recent preterm	80 (19.2)	240 (19.2)	
Most recent term	63 (15.1)	189 (15.1)	
≥3 Live births (last 3 deliveries)			
Most recent preterm	73 (17.5)	219 (17.5)	
Gestational diabetes^d^	49 (12.7)	109 (8.7)	.02
Severe features of preeclampsia^d^	39 (10.1)	128 (10.2)	.93

^a^ In the 3:1 match.

^b^ Includes Afgani, Arab (n = 2), Indian, Jordanian, Nepali, Pakastani, and unknown (n = 3).

^c^ Defined as a body mass index of 30 or higher (calculated as weight in kilograms divided by height in meters squared).

^d^ Denominator adjusted to 387 cases with data unavailable owing to delivering elsewhere.

The overall rate of recurrent PTB was 24.0% (100 of 417; 95% CI, 20.0%-28.4%) for the VP cohort compared with the 16.8% expected rate (1394 of 8278) in the matched historical Parkland obstetric population (Table 2; eFigure 1 in the Supplement). The unadjusted association for recurrent PTB measured by the OR of cases vs controls was 1.6 (95% CI, 1.2-2.1), and the adjusted for matched case-control was 1.8 (95% CI, 1.3-2.4), with ORs greater than 1 indicating higher rates of recurrent PTB in cases vs controls. We next analyzed each pregnancy according to specific obstetric history (Table 2). Regardless of prior PTB number or sequence, VP treatment was not associated with reduced rates of recurrence.

**Table 2.** Rate of Recurrent Preterm Birth Among Patients Treated With VP Compared With 3:1 Matched Historical Controls and Patients Receiving 17OHP-C for Preterm Birth History

Prior birth ≤35 wk	Historical, No./total No. (%)	17OHP-C, No./total No. (%)	Total No. of patients	No. of patients treated with VP delivering at ≤35 wk	Rate, % (95% CI)
Overall	1394/8278 (16.8)	106/430 (24.7)	417	100	24.0 (20.0-28.4)
1 Live birth	790/4490 (17.6)	44/141 (31.2)	122	34	27.9 (20.1-36.7)
2 Live birth					
Both ≤35 wk	155/362 (42.5)	20/48 (41.7)	32	1	34.4 (18.6-53.2)
Only second ≤35 wk	179/1081 (16.7)	11/52 (21.2)	48	11	22.9 (12.0-37.3)
Only first ≤35 wk	213/2001 (10.6)	2/39 (5.1)	63	11	17.5 (9.1-29.1)
≥3 Live births					
All ≤35 wk	20/44 (45.5)	12/27 (44.4)	16	8	50.0 (24.7-75.3)
Other sequence of ≤35 wk	37/300 (12.3)	17/123 (13.8)	136	25	18.4 (12.3-25.9)

Abbreviations: 17OHP-C, 17-alpha hydroxyprogesterone caproate; VP, vaginal progesterone.

We then analyzed recurrent PTB in the context of patient adherence (Table 3) and found that adequate adherence was not associated with lower rates of recurrent PTB (OR, 0.9 [95% CI, 0.5-1.4]). There was also no difference noted for any sequence of PTB history associated with adherence. Adherence with VP treatment was 34.3% (143 of 417); 43.6% of patients (182 of 417) recorded 70% or greater adherence, and 56.1% of patients (234 of 417) recorded 50% or greater adherence. Analysis did not demonstrate a reduction of recurrent PTB when examined according to adherence rates of either 70% or 50% (eTable 1 in the Supplement). Eighty percent of patients (52 of 65) discontinued medication because of reported adverse effects of discharge (24.6% [16 of 65]), irritation (23.1% [15 of 65]), pain (16.9% [11 of 65]), and itching (15.4% [10 of 65]). A total of 94 of 417 patients (22.5%) discontinued medication within 4 weeks of initiation (eFigure 2 in the Supplement). Despite medication nonadherence, the frequency of completed patient visits for those in the VP cohort was significantly greater than those with other high-risk pregnancy conditions (median number of visits, 13 [IQR, 10-16] vs 11 [IQR, 8-13]; *P* < .001) (eFigure 3 in the Supplement).

**Table 3.** Rate of Recurrent Preterm Birth Among Patients Adherent With Vaginal Progesterone Compared With Nonadherent Patients for Preterm Birth History

Prior birth ≤35 wk	No./total No. (%)		*P* value^a^	OR (95% CI)
	Adherent	Noncadherent		
Overall	32/143 (22.4)	68/274 (24.8)	.58	0.87 (0.51-1.41)
1 Live birth	8/45 (17.8)	26/77 (33.8)	.06	0.42 (0.17-1.04)
2 Live births				
Both ≤35 wk	4/14 (28.6)	7/18 (38.9)	.54	0.63 (0.14-2.81)
Only second birth ≤35 wk	3/15 (20.0)	8/33 (24.2)	.75	0.78 (0.18-3.48)
Only first birth ≤35 wk	5/24 (20.8)	6/39 (15.4)	.58	1.45 (0.39-5.39)
≥3 Live births				
All ≤35 wk	3/5 (60.0)	5/11 (45.5)	.59	1.80 (0.21-15.41)
Other sequences of ≤35 wk	9/40 (22.5)	16/96 (16.7)	.42	1.45 (0.58-3.63)
Cochran-Mantel-Haenszel test^b^	NA	NA	.50	0.54 (0.52-1.37)

Abbreviations: NA, not applicable; OR, odds ratio.

^a^ For comparison of adherent patients compared with nonadherent patients.

^b^ Adjustment for subset profile.

The change in gestational weeks of recurrent PTBs among patients treated with VP was then compared with the change in gestational weeks among patients previously untreated with progestogens but who delivered a recurrent PTB (Figure). The mean recurrent PTB before any progestogen was 0.4 weeks (95% CI, 0.2-0.7 weeks), and the mean recurrent PTB with VP treatment was 0.6 weeks (95% CI, −0.5 to 1.8 weeks). The mean difference between historical matched controls and those using VP was 0.2 weeks (95% CI, −1.4 to 1.0 weeks; *P* = .73). Thus, there was no improvement in the interval of recurrent PTB after the implementation of treatment with VP.

**Figure.**
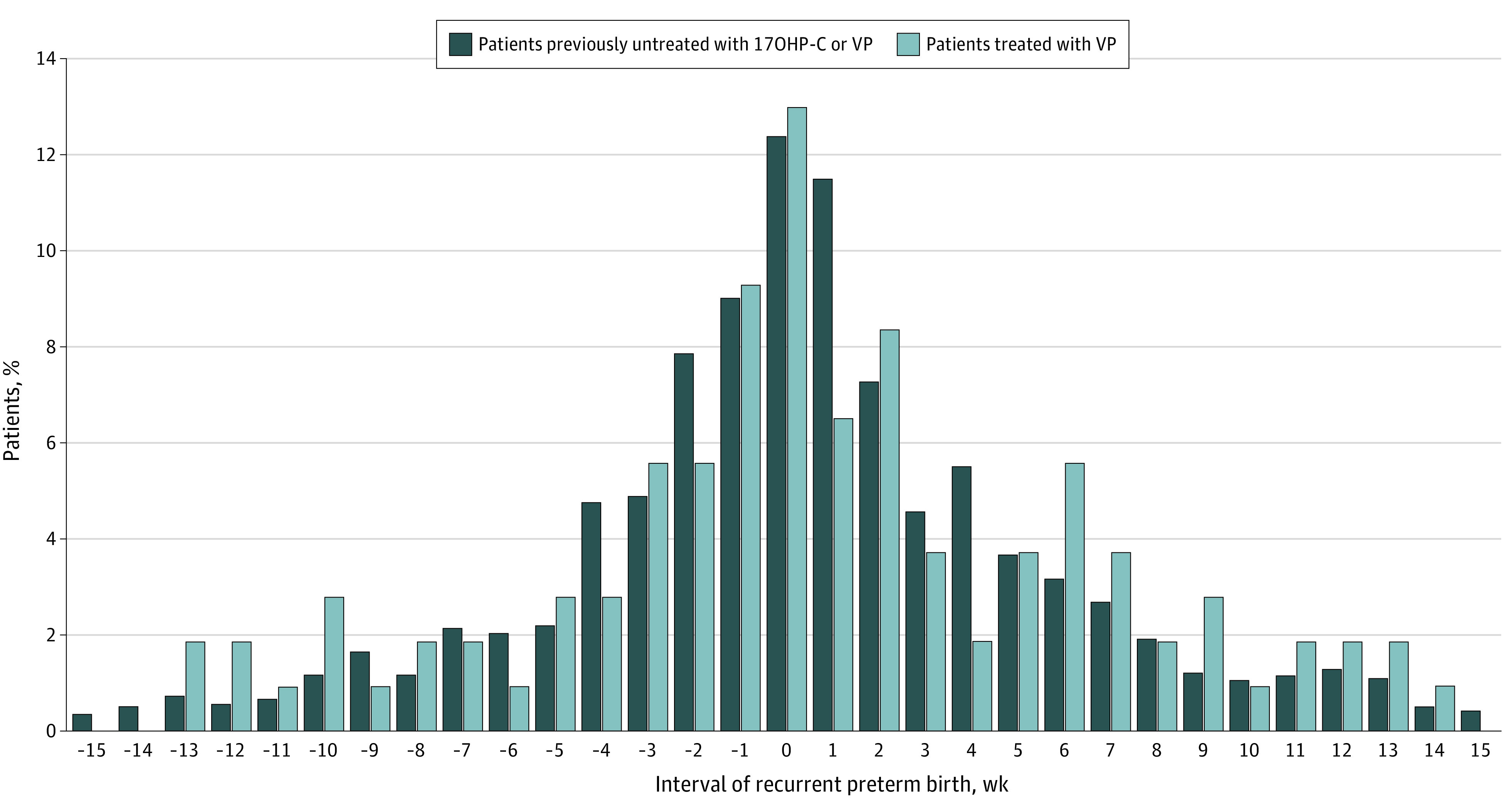
Recurrent Preterm Birth From Proximate Birth Among Patients Treated With Vaginal Progesterone (VP) Compared With Matched Controls 17OHP-C indicates 17-alpha hydroxyprogesterone caproate.

Progesterone levels were available for 186 patients at 24 weeks of gestation and 176 patients at 32 weeks of gestation. At 24 weeks, the plasma concentration of progesterone for those adherent with treatment (n = 73) was 99 ng/mL (95% CI, 85-121 ng/mL) (to convert progesterone to nanomoles per liter, multiply by 3.18), and the plasma concentration of progesterone for those nonadherent with treatment (n = 103) was 104 ng/mL (95% CI, 89-125 ng/mL) (*P* = .16). At 32 weeks, the plasma concentration of progesterone for those adherent with treatment (n = 74) was 200 ng/mL (95% CI, 171-242 ng/mL), and the plasma concentration of progesterone for those nonadherent with treatment (n = 94) was 196 ng/mL (95% CI, 155-271 ng/mL) (*P* = .69). Regression analysis is included in eFigure 4 in the Supplement.

Patients who declined therapy had a differing risk profile compared with those who received VP treatment (Table 4). The recurrence of PTB was compared among patients receiving VP treatment with those who declined to receive therapy during the contemporary period. Those who declined VP treatment had differing prematurity profiles, with higher parity and fewer index deliveries with PTB compared with current pregnancy. Last, patients treated with VP were compared with those who received 17OHP-C, with no significant differences noted in rates of PTB (OR, 1.03 [95% CI, 0.76-1.42]) (eTable 2 in the Supplement).

**Table 4.** Rate of Recurrent Preterm Birth Among Patients Treated With Vaginal Progesterone Compared With Patients Who Declined to Enroll in the Study for Preterm Birth History

Prior birth ≤35 wk	Patients who declined		Patients who enrolled		*P* value	OR (95% CI)
	Total, No.	≤35 wk, No. (%)	Total, No.	≤35 wk, No. (%)		
Overall	172	26 (15.1)	417	100 (24.0)	.02	0.56 (0.35-0.91)
1 Live birth	0	0	122	34 (27.9)	NA	NA
2 Live births						
Both ≤35 wk	9	6 (66.7)	32	11 (34.4)	.08	3.82 (0.80-18.28)
Only second birth ≤35 wk	0	0	48	11 (22.9)	NA	NA
Only first birth ≤35 wk	27	0	63	11 (17.5)	.02	0.08 (0.005-1.46)
≥3 Live births						
All ≤35 wk	5	4 (80.0)	16	8 (50.0)	.24	4.00 (0.36-44.11)
Other sequences of ≤35 wk	131	16 (12.2)	136	25 (18.4)	.16	0.62 (0.31-1.22)
Cochran-Mantel-Haenszel test^a^	NA	NA	NA	NA	.28	0.74 (0.43-1.27)

Abbreviations: NA, not applicable; OR, odds ratio.

^a^ Adjustment for subset profile.

## Discussion

The use of VP was not associated with a decrease in the overall rate of recurrent PTB when examined according to intention-to-treat analysis. In addition, the rates of PTB recurrence were not improved by VP treatment when analyzed according to the specific sequence of prior PTBs and term births. None of the a priori secondary analyses demonstrated that VP treatment was associated with a reduction in recurrent PTBs. First, improved adherence with VP treatment was not associated with reduced rates of recurrent PTB. Second, VP treatment was not associated with increased duration of pregnancy when patients with a recurrent PTB were compared with similar patients not previously treated with a progestogen. Third, plasma levels were not significantly different between those with and those without adherence with VP therapy, and blood levels did not demonstrate a significant difference in recurrent PTBs. Fourth, there were no significant reductions in PTBs among those treated with VP and those who declined therapy during the contemporary study period. Fifth, the rates of recurrent PTB were not different when comparing patients receiving VP with those receiving 17OHP-C.

Use of progestogens to prevent PTB has been explored for more than half a century. However, defining for whom, and if, progestogen use reduces PTB has yet to be clearly defined.^18^ Because of the challenges with 17OHP-C treatment, there has been renewed interest in using VP for patients with a prior PTB.^15,19^ A recent individual participant data meta-analysis of 31 randomized clinical trials evaluated the use of progestogens for the prevention of PTB among patients with varying risks, including prior spontaneous PTB and short cervix.^19^ These investigators found that VP treatment was associated with a reduced risk of recurrent early PTB (<34 weeks’ gestation) in singleton pregnancies compared with untreated controls (relative risk, 0.78 [95% CI, 0.68-0.90]). These investigators, however, were unable to determine whether PTB was spontaneous for all patients in all trials. Our findings differ from this report by evaluating only patients with spontaneous—rather than indicated (eg, preeclampsia)—PTB. When analyzing interventions to prevent recurrent spontaneous PTB, an individual patient’s medical history is critical. That is, attributing benefit associated with therapy must account for each individual’s risk profile, including both the prior number and sequence of PTBs and term births.^4,5^ Our findings are consistent, however, with another systematic review and meta-analysis.^20^ This report highlighted the variation in published reports wherein sensitivity analyses restricted to studies at low risk of bias indicated that VP treatment was not associated with a reduction in the risk of birth at less than 37 weeks and less than 34 weeks.^20^ We offer our current experience with VP treatment as further information to guide PTB prevention efforts.

### Limitations and Strengths

Our study had several limitations. The lack of generalizability from this single-center report is a limitation. Specifically, Parkland Hospital serves a medically indigent population composed predominantly of Hispanic and non-Hispanic Black patients with obesity. In addition, adherence with therapy was low; however, this report offers clinical experience with patient tolerance of the therapy.^21^ Indeed, 80% of patients discontinued therapy owing to reported adverse effects of medication. Moreover, levels of progesterone in the blood did not suggest a differing response to therapy. That is, there was no evidence of a dose response, or ability to measure adherence, based on the elevated physiological levels found in normal pregnancy. There is a suggestion, however, that use of VP may increase rates of recurrent PTB among this population. Alterations of vaginal flora and the microbiome do not appear to be altered by VP but may offer some explanation for these findings and are a future area of possible research.^22^ Short cervical length as an indication for VP treatment was beyond the scope of this analysis, and thus we cannot comment on clinical performance for this indication.^23^

There are also several strengths of this prospective study. First, the analysis used clinical data abstracted by trained research nurses rather than administrative data. Second, clinical outcomes were measured according to specific PTB risk profiles. Third, multiple dimensions of VP were explored to include progesterone levels, severity reduction, and comparison with prior time periods of 17OHP-C use.

## Conclusions

In this prospective cohort study of patients with current singleton pregnancies, VP treatment was not associated with a reduction in recurrent PTBs compared with matched historical controls for number and sequence of prior PTBs. It also was not associated with improved clinical outcomes associated with medication adherence, severity of recurrent PTB, or progesterone blood levels. These findings suggest that pregnant patients should not be offered VP to prevent recurrent PTB based on personal PTB history alone.

## Notes

Supplement. **eTable 1.** Rate of Recurrent Preterm Birth Among Patients Adherent With Vaginal Progesterone at 70%, 60%, and 50% Compared to Nonadherent Patients for Overall Recurrent Preterm and According to Specific Sequence of Prior Preterm Birth History **eTable 2.** Rate of Recurrent Birth ≤35 Weeks Among Patients With 17OHP-C Treated Pregnancies Compared to Those With Vaginal Progesterone (VP) **eFigure 1.** Frequency and Distribution of 3:1 Matched Historical Cohort to VP Cohort for Black Race, Obesity, and Specific Preterm Birth Profile From 1998-2011 **eFigure 2.** Frequency Distribution of the Week of the Patient’s Last VP Dose **eFigure 3.** Number of Prenatal Clinic Visits (Median [1^st^ Quartile, 3^rd^ Quartile]) for VP Cohort Compared to General Obstetric Population **eFigure 4.** Relationship Between Adherence and Blood Levels in Patients at Both 24 and 32 Weeks as a Regression Analysis
