# Supplementary material for: Association of Vaginal Progesterone Treatment With Prevention of Recurrent Preterm Birth
Source: JAMA Netw Open. 2022 Oct 31;5(10):e2237600. doi: 10.1001/jamanetworkopen.2022.37600 (PMC9623441; doi:10.1001/jamanetworkopen.2022.37600)
Supplement: Supplement. — eTable 1. Rate of Recurrent Preterm Birth Among Patients Adherent With Vaginal Progesterone at 70%, 60%, and 50% Compared to Nonadherent Patients for Overall Recurrent Preterm and According to Specific Sequence of Prior Preterm Birth History eTable 2. Rate of Recurrent Birth ≤35 Weeks Among Patients With 17OHP-C Treated Pregnancies Compared to Those With Vaginal Progesterone (VP) eFigure 1. Frequency and Distribution of 3:1 Matched Historical Cohort to VP Cohort for Black Race, Obesity, and Specific Preterm Birth Profile From 1998-2011 eFigure 2. Frequency Distribution of the Week of the Patient’s Last VP Dose eFigure 3. Number of Prenatal Clinic Visits (Median [1st Quartile, 3rd Quartile]) for VP Cohort Compared to General Obstetric Population eFigure 4. Relationship Between Adherence and Blood Levels in Patients at Both 24 and 32 Weeks as a Regression Analysis [file jamanetwopen-e2237600-s001.pdf]

Supplementary Online Content

Nelson DB, Lafferty A, Venkatraman C, et al. Association of vaginal progesterone treatment with prevention of recurrent preterm birth. *JAMA Netw Open.* 2022;5(10):e2237600. doi:10.1001/jamanetworkopen.2022.37600

eTable 1. Rate of Recurrent Preterm Birth Among Patients Adherent With Vaginal Progesterone at 70%, 60%, and 50% Compared to Nonadherent Patients for Overall Recurrent Preterm and According to Specific Sequence of Prior Preterm Birth History
eTable 2. Rate of Recurrent Birth ≤35 Weeks Among Patients With 17OHP-C Treated Pregnancies Compared to Those With Vaginal Progesterone (VP)
eFigure 1. Frequency and Distribution of 3:1 Matched Historical Cohort to VP Cohort for Black Race, Obesity, and Specific Preterm Birth Profile From 1998-2011
eFigure 2. Frequency Distribution of the Week of the Patient's Last VP Dose
eFigure 3. Number of Prenatal Clinic Visits (Median [1st Quartile, 3rd Quartile]) for VP Cohort Compared to General Obstetric Population
eFigure 4. Relationship Between Adherence and Blood Levels in Patients at Both 24 and 32 Weeks as a Regression Analysis

This supplementary material has been provided by the authors to give readers additional information about their work.

© 2022 Nelson DB et al. *JAMA Network Open.*

eTable 1. Rate of Recurrent Preterm Birth Among Patients Adherent With Vaginal Progesterone at 70%, 60%, and 50% Compared to Nonadherent Patients for Overall Recurrent Preterm and According to Specific Sequence of Prior Preterm Birth History

	Adherent	Nonadherent	P-value
Prior birth ≤ 35wk			
Overall at 70%	37/182 (20.3)	63/235 (26.8)	0.12
Overall at 60%	44/207 (21.2)	56/210 (26.7)	0.20
Overall at 50%	55/234 (23.5)	45/183 (24.6)	0.80

Data presented as N/total (%).
P-values are two-sided tests

© 2022 Nelson DB et al. *JAMA Network Open.*

eTable 2. Rate of Recurrent Birth ≤35 Weeks Among Patients With 17OHP-C Treated Pregnancies Compared to Those With Vaginal Progesterone (VP)

	17OHP-C	Vaginal Progesterone	P-value	OR (95% CI)
Prior birth ≤35 weeks				
Overall	106/430 (24.7)	100/417 (24.0)	0.82	1.03 (0.76, 1.42)
Para 1	44/141 (31.2)	34/122 (27.9)	0.55	1.17 (0.69, 2.00)
Para 2				
Both ≤35 weeks	20/48 (41.7)	11/32 (34.4)	0.51	1.36 (0.54, 3.45)
Only 2nd birth ≤35 weeks	11/52 (21.2)	11/48 (22.9)	0.83	0.90 (0.35, 2.33)
Only 1st birth ≤35 weeks	2/39 (5.1)	11/63 (17.5)	0.07	0.26, (0.05, 1.22)
Para ≥3				
All ≤35 weeks	12/27 (44.4)	8/16 (50.0)	0.72	0.80 (0.23, 2.76)
Other sequence of ≤35 weeks	17/123 (13.8)	25/136 (18.4)	0.32	0.71 (0.36, 1.39)
Cochran-Mantel-Haenszel			0.58	0.91 (0.66, 1.26)

Data shown as N/total (%).

© 2022 Nelson DB et al. *JAMA Network Open.*

eFigure 1. Frequency and Distribution of 3:1 Matched Historical Cohort to VP Cohort for Black Race, Obesity, and Specific Preterm Birth Profile From 1998-2011

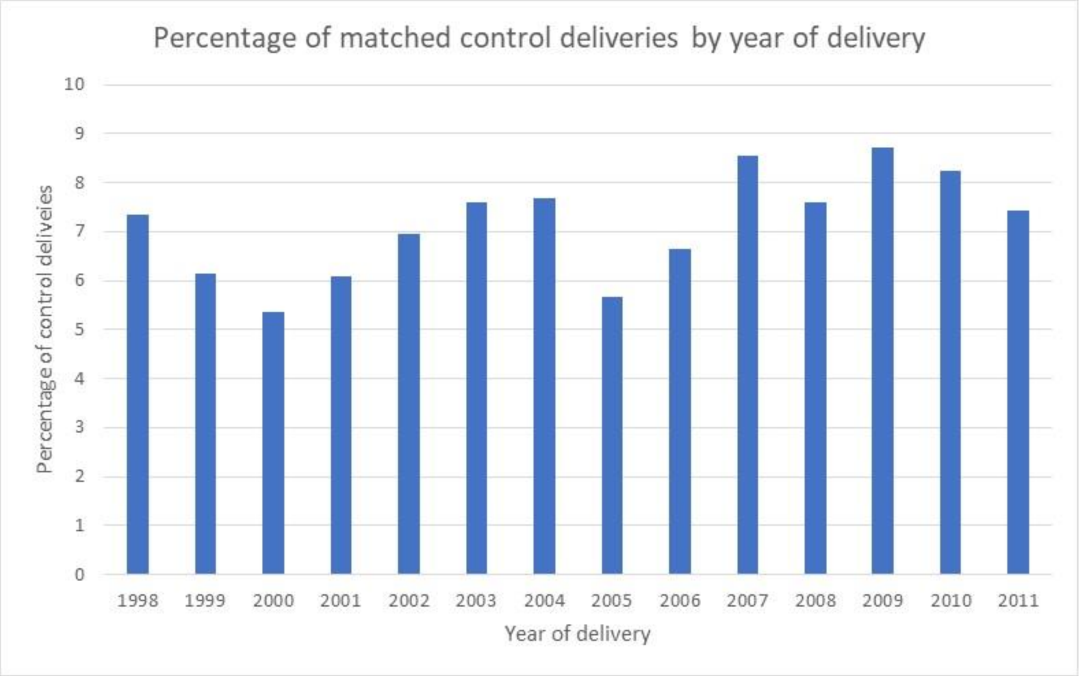

© 2022 Nelson DB et al. *JAMA Network Open.*

eFigure 2. Frequency Distribution of the Week of the Patient's Last VP Dose

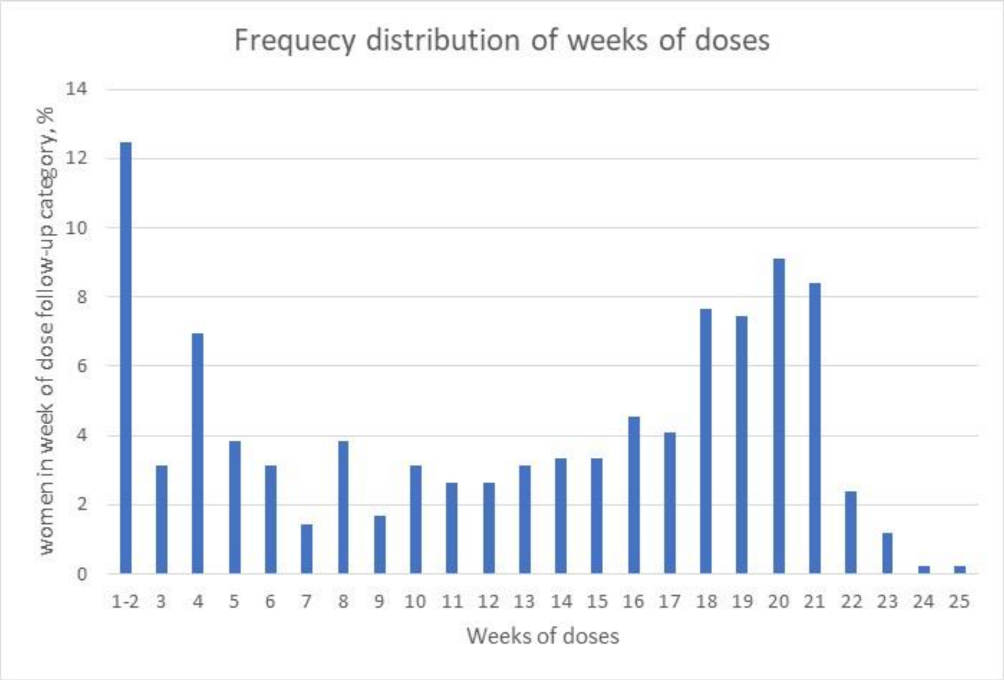

© 2022 Nelson DB et al. *JAMA Network Open.*

eFigure 3. Number of Prenatal Clinic Visits (Median [1st Quartile, 3rd Quartile]) for VP Cohort Compared to General Obstetric Population. P<0.001, Wilcoxon rank-sum test.

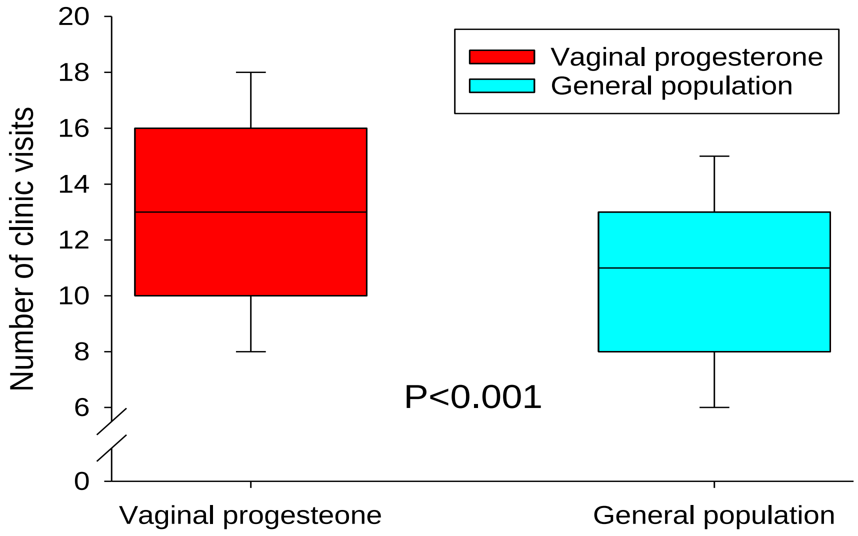

© 2022 Nelson DB et al. *JAMA Network Open.*

eFigure 4. Relationship Between Adherence and Blood Levels in Patients at Both 24 and 32 Weeks as a Regression Analysis

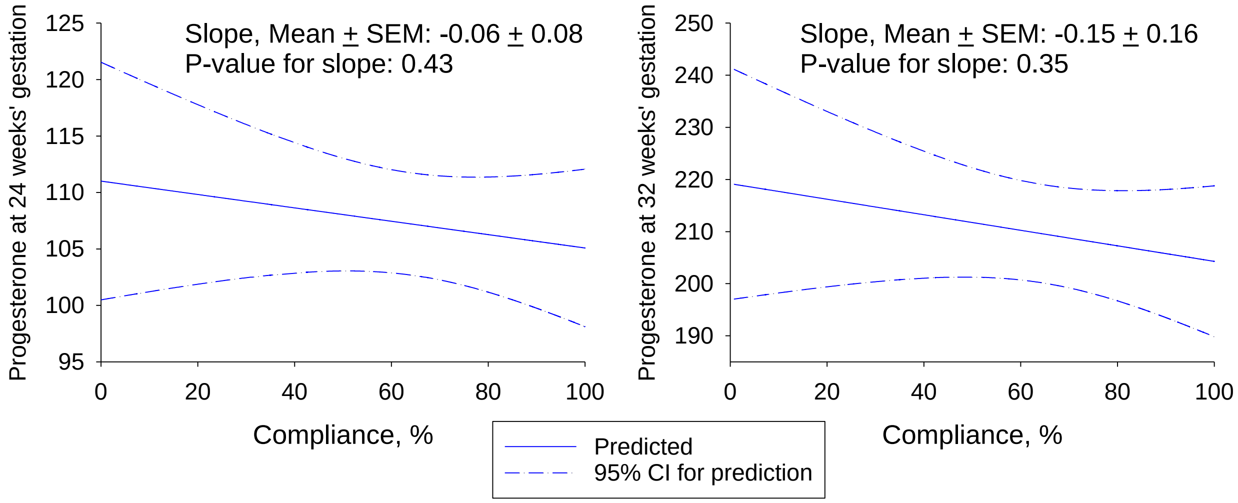

© 2022 Nelson DB et al. *JAMA Network Open.*
